# The regulatory mechanism of HSP70 in endoplasmic reticulum stress in pepsin-treated laryngeal epithelium cells and laryngeal cancer cells

**DOI:** 10.18632/aging.204356

**Published:** 2022-10-27

**Authors:** Wei Chen, Zhiyi Wang, Junfeng Ji, Tao Shi, Hong ye Jiao, You Cheng, Li Xu, Rui Wang

**Affiliations:** 1 Department of Otolaryngology-Head and Neck, Jinling Hospital, Medical College of Nanjing University, Nanjing 210002, Jiangsu, China; 2 Department of Otolaryngology-Head and Neck, The First School of Clinical Medicine, Southern Medical University, Nanjing 210002, Jiangsu, China; 3 Department of Medical Oncology, Jinling Hospital, Medical College of Nanjing University, Nanjing 210002, Jiangsu, China

**Keywords:** HSP70, endoplasmic reticulum stress, laryngeal cancer, apoptosis, autophagy

## Abstract

Backgrounds: Excessive pepsin can damage both normal laryngeal epithelial cells and laryngeal cancer (LC) cells. Heat shock protein 70 (HSP70) is closely related to pepsin. In this paper, we will explore the different significance of the regulatory role of HSP70 in endoplasmic reticulum stress (ERS) level in pepsin-treated laryngeal epithelial cells and LC cells.

Methods: In cell experiments, laryngeal epithelial cells and LC cells were selected and induced by different concentrations of pepsin. Cell activity was detected by CCK8, cell apoptosis was detected by flow cytometry, and autophagy was detected by autophagy detection kit. The expression of ER)-related proteins was detected by immunofluorescence (IF) and Western blot. Cell transfection was used to inhibit HSP70 expression in both cells, and ERS, apoptosis, and autophagy were measured using related techniques. In animal experiments, a mouse model bearing LC was established. TUNEL assay detected apoptosis, autophagy kit detected autophagy, and ER-related protein expression was detected by Western blot.

Results: HSP70 was increased in pepsin-stimulated laryngeal epithelial cells and LC cells, thereby inhibiting ER and ER-induced apoptosis and autophagy. Inhibition of HSP70 reduced the expression of glucose regulated protein 78 (GRP78) in pepsin-stimulated laryngeal epithelial cells and LC cells, and only inhibited downstream apoptosis-related pathways in laryngeal epithelial cells rather than in LC cells. Inhibition of HSP70 and ER could significantly promote apoptosis and inhibit tumor growth in the absence of pepsin stimulation *in vivo*.

Conclusion: ER level regulated by HSP70 had different significance in laryngeal epithelial cells and LC cells treated with pepsin.

## INTRODUCTION

Laryngeal cancer (LC) is one of the commonest malignant tumors in otorhinolaryngology, more than 90% of which is squamous cell carcinoma [1]. The incidence of LC has been increasing year by year in recent years. Therefore, it is of great significance to explore the mechanism of occurrence and development of LC to find new and more effective diagnosis and treatment strategies of LC.

Laryngopharyngeal reflux (LPR) refers to the reflux of gastric contents to the upper esophageal sphincter above the site, causing a series of laryngopharyngeal symptoms and signs [2]. In recent years, the incidence rate of LPR in LC patients is above 50% [3]. Gastric acid stimulates laryngeal mucosa repeatedly along with reflux, causing inflammation and injury, thus causing pre-cancerous lesions and cancer [4]. LPR is an important factor promoting the development of LC. Therefore, the mechanism of LPR lesions at different stages is discussed in this study. Pepsin is the main component of gastroesophageal reflux, and it is an indispensable proteolytic enzyme in digestive system [5]. Clinical studies have shown that pepsin is highly expressed in tumor tissues of patients with LC [6, 7]. Pepsin can be taken up by laryngeal epithelial cells through the corresponding receptors, thus changing the expression of acid-mediated stress proteins, and leading to the aggravation of a series of pathological changes [8]. Therefore, the mechanism of pepsin in different stages of laryngeal lesions is discussed in this paper.

HSP70 is one of the most major chaperone proteins, which can be induced by organisms to overcome stress under stress conditions. Therefore, HSP70 is also called a stress protein [9]. HSP70 can perform many biological functions, including facilitating the folding and assembly of newly synthesized proteins, refolding of misfolded and aggregated proteins, regulating the movement of organelles and membrane of secreted proteins, and controlling and regulating the activity of proteins [10, 11]. A previous study has shown that HSP70 is a protein significantly overexpressed in LPR [12]. The increased expression of HSP70 can boost gastric mucosal blood flow and enhance gastric mucosal resistance to injury stimulation [13]. Further studies have shown that pepsin induces a significant increase of HSP70 even at pH7.0, which at least indicates that the increase of HSP70 is not dependent on gastric acid stimulation, but is closely related to the effect of pepsin [14, 15].

HSP70 is rapidly expressed in a variety of stress states, such as unfolded protein response (UPR) induced by the accumulation of misfolded proteins, and endoplasmic reticulum stress (ERS). Although some researchers have linked inflammation, precancerous lesions with the high expression of HSP70 and considered HSP70 as a marker, more views believe that from the perspective of the mechanism of action, the increase of HSP70 actually protects against the stress in the body and cells, which may involve the multifaceted regulation of HSP70 on ERS, autophagy and apoptosis [16].

The transformation of normal cells into cancer cells is stimulated by a variety of factors, resulting in changes in the property and function of cells caused by gene mutations. As a result, the role of HSP70 is likely to change significantly during this process. Therefore, ERS and HSP70 should be significantly different at different stages of LPR lesions. Based on the above background, this study further explores how the ERS level represented by HSP70 affects the laryngeal epithelium and LC cells.

## MATERIALS AND METHODS

### Culture of rat primary laryngeal epithelial cells

Rat throat epithelial tissues were cut into fragments and then were put into 1.5 mL centrifuge tube. Dispersive enzyme (Sigma) were added and soaked at 4°C overnight. Next day, the tissues were centrifuged at 1500 rpm for 5 min, the supernatant was discarded, and trypsin was added for digestion for 5 min at 37°C. Then culture medium was added to stop digestion and the tissue was repeatedly blown with the tip to form a single cell suspension. Next, cell suspension was filtered through a 10 μm screen. The filtrate was centrifuged at 1000 rpm for 5 min, and the supernatant was discarded. The bottom cells were re-suspended and cultured in DMEM (Invitrogen, Carlsbad, CA, USA) with 10% FBS (HyClone, Logan, UT, USA) and 1% penicillin/ Streptomycin (Invitrogen). At about 14 days, the cells were subcultured.

### Cell culture

Laryngeal epithelial cells and laryngeal cancer cells AMC-HN-8 were maintained in DMEM (Invitrogen, Carlsbad, CA, USA) with 10% FBS and 1% penicillin/streptomycin at 37°C, 5% CO_2_. 0.1 and 1 mg/ml pepsin were added in the medium (pH7.0), and the two cells were divided into control group, 0.1 and 1 mg/ml pepsin group, respectively.

### Flow cytometry

Apoptosis was detected with Annexin V-FITC Apoptosis Detection Kit II (BD Bioscience; San Jose, CA, USA) according to the manufacturer’s instructions. Cells were then analyzed by a FACSCalibur flow cytometer (BD Bioscience). The data were analyzed using WinMDI software (The Scripps Research Institute, La Jolla, CA, USA).

### Detection of autophagy

Autophagy vacuoles were detected with an Autophagy Detection kit (cat. no. ab139484, Abcam) according to the manufacturer’s protocol. Cells were seeded (2 × 10^4^ cells/well) in the 24-well plate. Cells or mouse tumor tissue were fixed with 4% cold paraformaldehyde for 15 min and then blocked with 1% BSA (Sigma-Aldrich; Merck KGaA) for 30 min at room temperature. The fluorescent dyes for nuclei staining and autophagy detection were added and incubated for 30 min at room temperature. Finally, the slides were observed using a confocal laser scanning microscope (FV1000s-SIM/IX81, Olympus Corporation).

### Immunofluorescence staining (IF)

Normal cells and AMC-HN-8 cells induced by pepsin were fixed by 4% paraformaldehyde at 25°C for 1 h, followed by permeabilization by 0.1% Triton X-100 for 5 min. Cells were blocked by 5% BSA for 30 min and incubated with primary antibodies against GRP78 (Cat#ab21685; Abcam) for 2 h. Following probing with secondary antibodies for 1 h at 37°C, cells were observed by fluorescence microscope (Olympus).

### Western blot

Proteins (30 μg) were subjected to sodium dodecyl sulfate polyacrylamide gel electrophoresis and then transferred to the PVDF membrane (Millipore). The membrane was blocked with 5% nonfat milk and incubated with the primary antibody overnight at 4°C. Next day, the membranes were conjugated with secondary antibody. Blots were detected with ECL detection reagent (Pierce, Rockford, IL, USA). The images were analyzed using ImageJ software (version 1.46).

### Cell transfection

Sh-NC, shRNA-HSP70#1 and shRNA-HSP70#2 stable interference plasmids were generated as standard. Briefly, shRNAs were directly ligated into pLKO.1 vector. Then, 1 mg of pLKO.1-shRNA, 0.5 mg of pPAX2, 0.5 mg of pVSVG were introduced into normal cells and AMC-HN-8 cells and cultured for 36 h. Lentivirus were collected and concentrated. We used lentivirus to infect cells for additional 24 h. To obtain stable cells, 2 mg/mL of puromycin was utilized to treat the cells.

### Mouse model bearing LC

20 BLAB/c nude mice (5 mice each group) were subcutaneously injected with a million of AMC-HN-8 cells. Mice were intraperitoneally injected with HSP70 activator ML346, HSP70 inhibitor VER-155008 and ERS inhibitor 4-PBA. The tumor-bearing mice were divided into control, ML346, VER-155008 and 4-PBA groups. About 28 days later, the mice were terminally sacrificed and tumors were removed. Relevant tissues were removed for reserve and tumor size and weight were calculated. Our research protocols were approved by ethics committee of Jinling Hospital.

### TUNEL assay

The TdT-UTP nick end labelling (TUNEL) assay was used to detect apoptosis with a TUNEL assay kit (Roche Diagnostics GmbH, Germany) according to the manufacturer’s instructions. Briefly, the tumor tissues were dewaxed and dehydrated conventionally. They were then incubated with TUNEL reagents containing terminal deoxynucleotidyl transferase (TdT) and fluorescent isothiocyanate dUTP for 2 hours at 37°C. At last, the samples were stained with DAPI for 30 minutes. The apoptotic cells were recognized with dual TUNEL and DAPI staining under a fluorescence and UV light microscope.

### Statistical analysis

Data were analyzed using Prism 5 software (GraphPad, La Jolla, CA, USA). Experimental data are expressed as means ± standard deviations. Differences between two or more groups were estimated using a Student *t* test or one-way analysis of variance (ANOVA). A *P* value of <0.05 was considered statistically significant.

### Consent for publication

All the authors agreed to be published.

### Availability of data and materials

The analyzed data sets generated during the present study are available from the corresponding author on reasonable request.

## RESULTS

### Pepsin treatment promoted apoptosis, autophagy and ERS and enhanced HSP70 expression in laryngeal epithelial cells and LC cells and laryngeal epithelial cells were more sensitive to pepsin stimulation than LC cells

**Figure 1:**
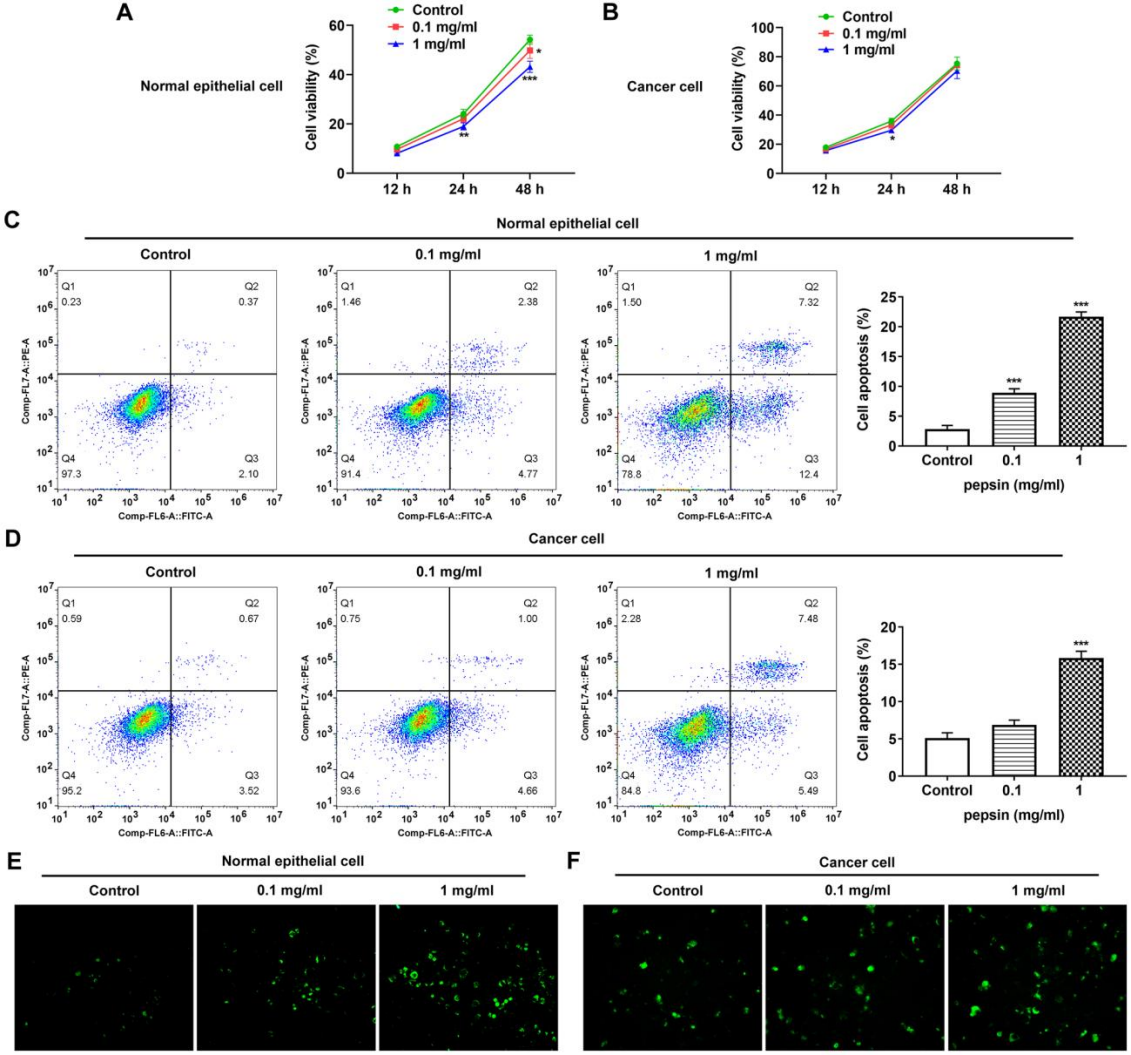
**Pepsin inhibited viability while promoted apoptosis and autophagy in laryngeal epithelial cells and LC cells.** (**A** and **B**) CCK8 detected the viability in laryngeal epithelial cells and LC cells. (**C** and **D**) Apoptosis of laryngeal epithelial cells and LC cells were detected by flow cytometry. (**E** and **F**) The autophagy detection kit measured autophagy levels. ^*^p < 0.05, ^**^p < 0.01, ^***^p < 0.001 vs. Control.

**Figure 2:**
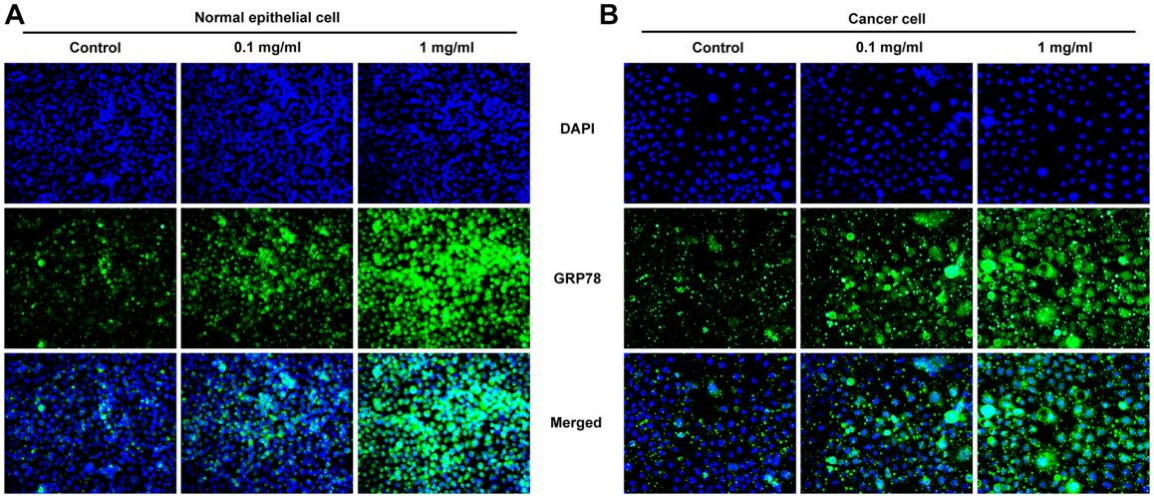
**Pepsin increased the expression of GRP78 in laryngeal epithelial cells and LC cells.** (**A** and **B**) IF assay was used to detect the expression of ERS-related protein GRP78 in laryngeal epithelial cells and LC cells. ^*^p < 0.05, ^**^p < 0.01, ^***^p < 0.001 vs. Control.

**Figure 3:**
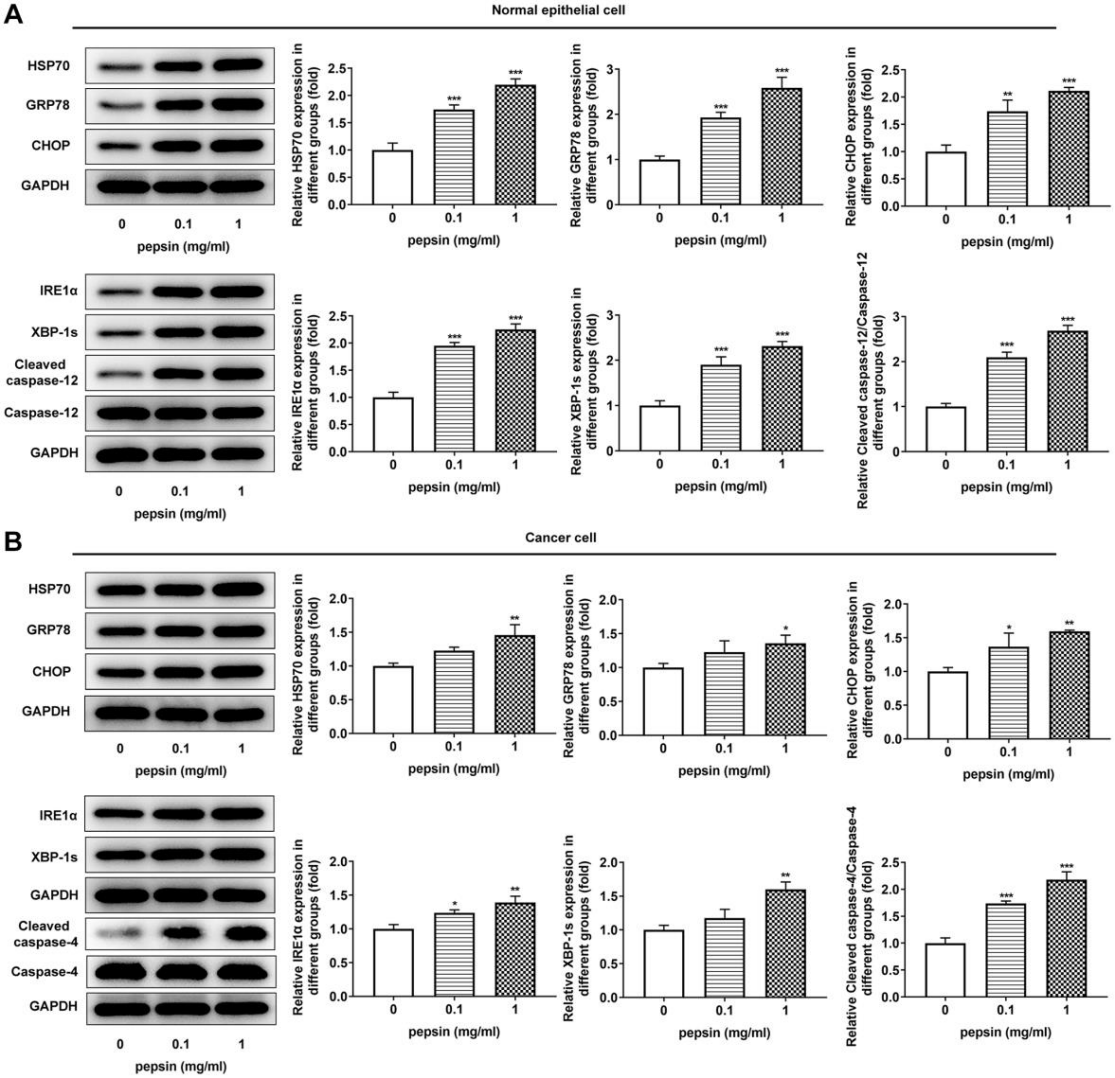
**Pepsin enhanced the expression of HSP70 and ERS-related proteins in laryngeal epithelial cells and LC cells.** (**A** and **B**) Western blot detected the expression of ERS-related proteins in laryngeal epithelial cells and LC cells. ^*^p < 0.05, ^**^p < 0.01, ^***^p < 0.001 vs Control.

After inducing laryngeal epithelial cells and LC cells with different concentrations of pepsin, the cell activity was detected by CCK8. Results showed that normal cell activity was significantly decreased in 0.1 mg/ml and 1 mg/ml pepsin groups compared with the control group (Figure 1A and 1B). Flow cytometry showed that apoptosis of normal cells and cancer cells was significantly increased in 0.1 mg/ml and 1 mg/ml pepsin groups compared with the control group (Figure 1C and 1D). The results indicated that pepsin caused greater damage on normal cells more than cancer cells. Subsequently, autophagy levels were detected by the autophagy kit, and the results showed that autophagy levels of both normal and cancer cells were significantly increased after pepsin induction compared with the control group (Figure 1E and 1F). IF detected the expression of ERS-associated protein GRP78, and we found that the expression of GRP78 in normal cells and cancer cells was significantly increased in 0.1 mg/ml and 1 mg/ml pepsin groups compared with the control group (Figure 2A and 2B). The expression of ERS-related proteins and HSP70 was detected by Western blot, and it was found that the expression of HSP70 was significantly increased after pepsin induction. The expression of ERS-related proteins GRP78, CHOP, IRE1α, XBP-1s, cleaved-caspase-12 and cleaved caspase 4 were also significantly increased in normal cells and cancer cells when treated by 0.1 mg/ml and 1 mg/ml pepsin (Figure 3A and 3B).

### Silencing of HSP70 inhibited the viability while stimulated the apoptosis of pepsin-treated laryngeal epithelial cells and LC cells, and only suppressed GRP78 expression in laryngeal epithelial cells instead of in LC cells

**Figure 4:**
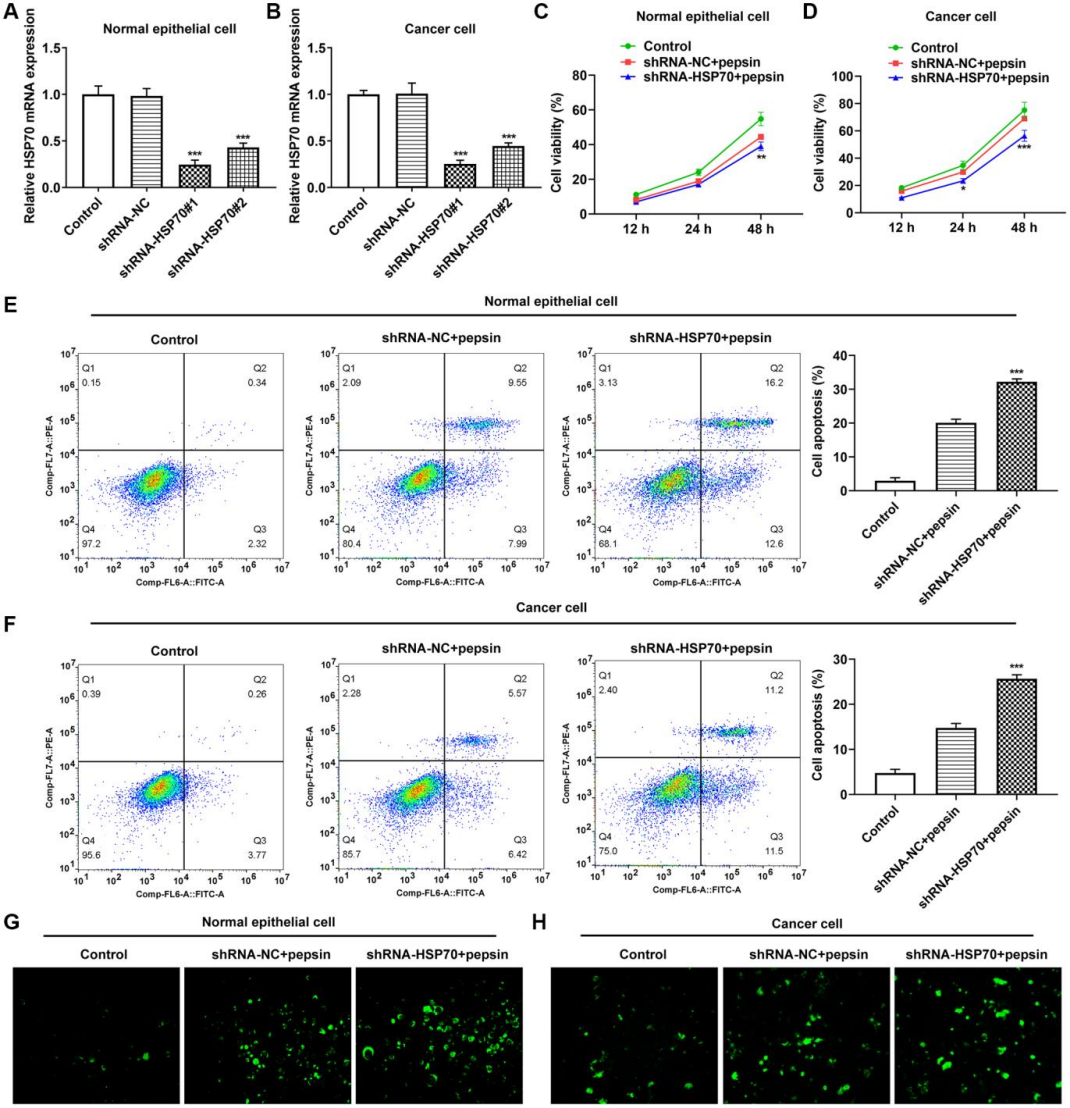
**The effect of inhibition of HSP70 on cell viability, apoptosis and autophagy.** (**A** and **B**) RT-qPCR detected the expression of HSP70 in laryngeal epithelial cells and LC cells. ^***^p < 0.001 vs. shRNA-NC. (**C** and **D**) CCK8 detected the viability in laryngeal epithelial cells and LC cells. (**E** and **F**) Apoptosis of laryngeal epithelial cells and LC cells were detected by flow cytometry. (**G** and **H)** The autophagy detection kit measured autophagy levels in laryngeal epithelial cells and LC cells. ^**^p < 0.01, ^***^p < 0.001 vs shRNA-NC+pepsin.

**Figure 5:**
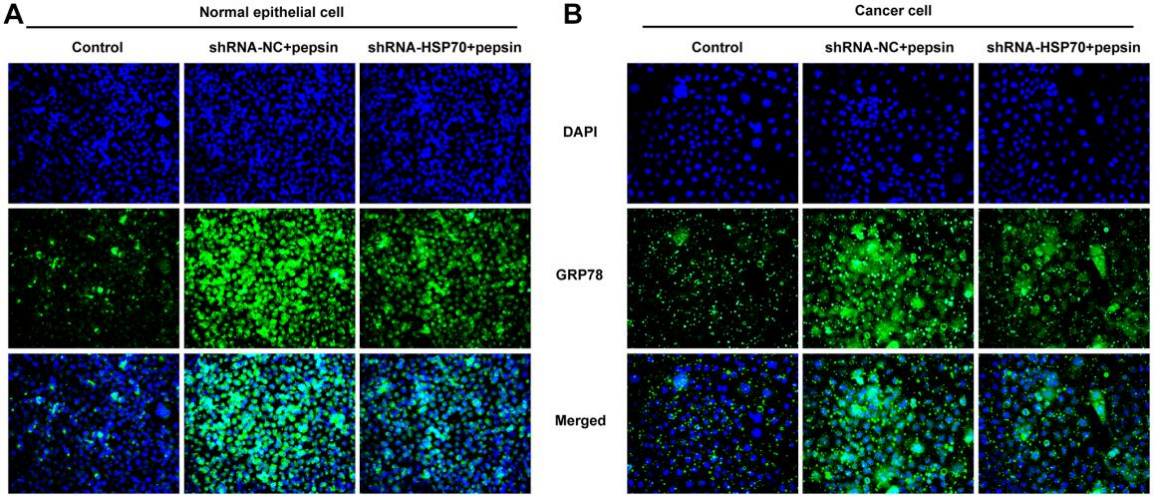
**HSP70 silencing decreased the expression of GRP78 in pepsin-stimulated laryngeal epithelial cells and LC cells.** (**A** and **B**) IF assay was used to detect the expression of ERS-related protein GRP78 in laryngeal epithelial cells and LC cells.

**Figure 6:**
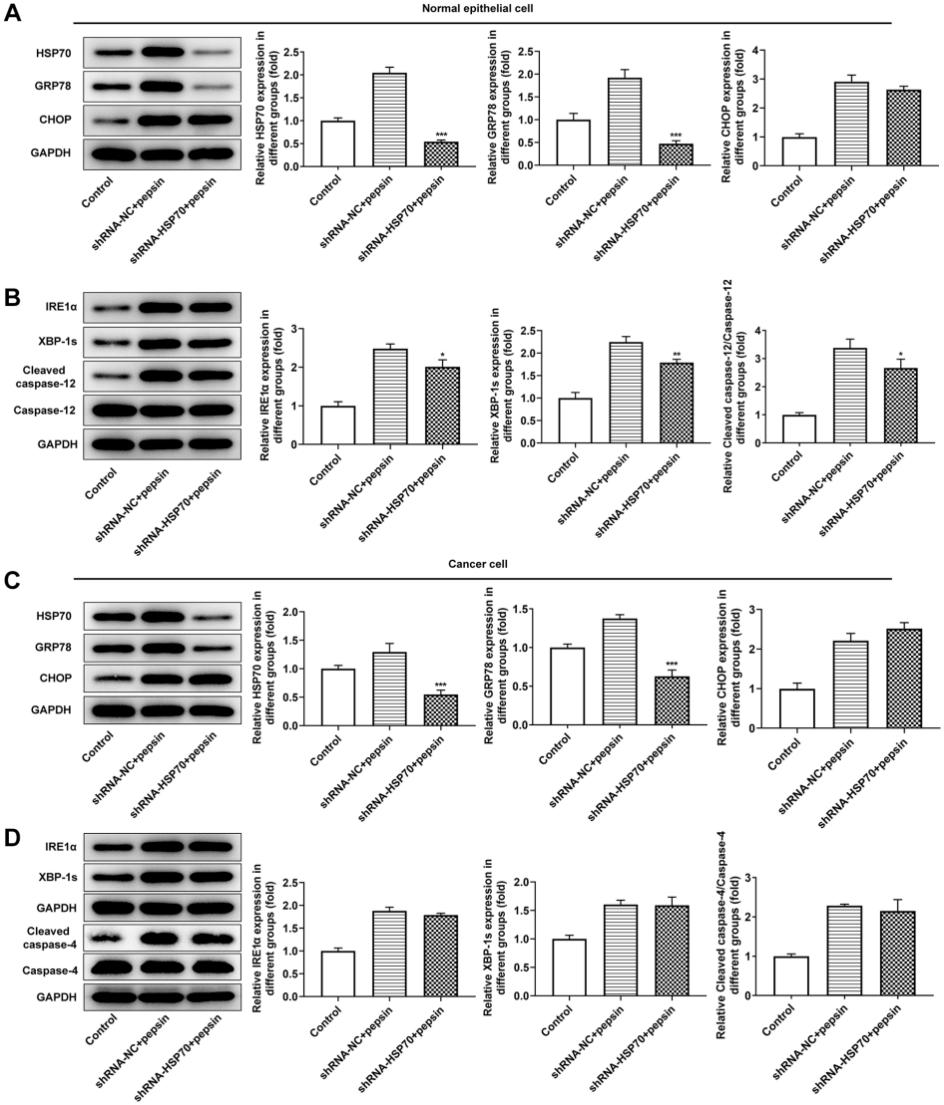
**Inhibition of HSP70 only inhibited downstream apoptosis-related pathways in laryngeal epithelial cells instead of in LC cells.** (**A** and **B**) Western blot detected the expression of ERS-related proteins in laryngeal epithelial cells. (**C** and **D**) Western blot detected the expression of ERS-related proteins in LC cells. ^*^p < 0.05, ^**^p < 0.01, ^***^p < 0.001 vs. shRNA-NC+pepsin.

After HSP70 was silenced in normal and LC cells, HSP70 expression was tested. The results uncovered that HSP70 expression was remarkably lessened after transfection of shRNA-HSP70#1/2 plasmids and shRNA-HSP70#1 displayed a more excellent interference efficiency, thereby being chosen for the subsequent experiments (Figure 4A and 4B). Then normal and LC cells were grouped into control, shRNA-NC+pepsin and shRNA-HSP70+pepsin. Compared with shRNA-NC+ pepsin group, inhibition of HSP70 expression significantly reduced cell activity (Figure 4C and 4D). After HSP70 was knocked down in pepsin-treated normal epithelial cells and LC cells, apoptosis was significantly increased (Figure 4E and 4F), autophagy significantly increased (Figure 4G and 4H). Subsequently, IF detected the expression of ERS-associated protein GRP78 (Figure 5A and 5B) and Western blot detected the expression of ERS-related proteins and HSP70 (Figure 6A and 6B). It was found that HSP70, GRP78, IREα, XBP-1S and cleaved caspase-4 of shRNA-HSP70+pepsin group were significantly lower than those of shRNA-NC+pepsin group in normal cells. The expression of CHOP showed no significant change. However, in LC cells, compared with shRNA-NC+pepsin group, the expression of HSP70 and GRP78 distinctly were decreased in shRNA-HSP70+pepsin group, but there was no significant change in the expression of other ERS-related proteins (Figure 6C and 6D).

### Inhibition of HSP70 and ERS could significantly promote apoptosis and inhibit tumor growth in the absence of pepsin stimulation *in vivo*

**Figure 7:**
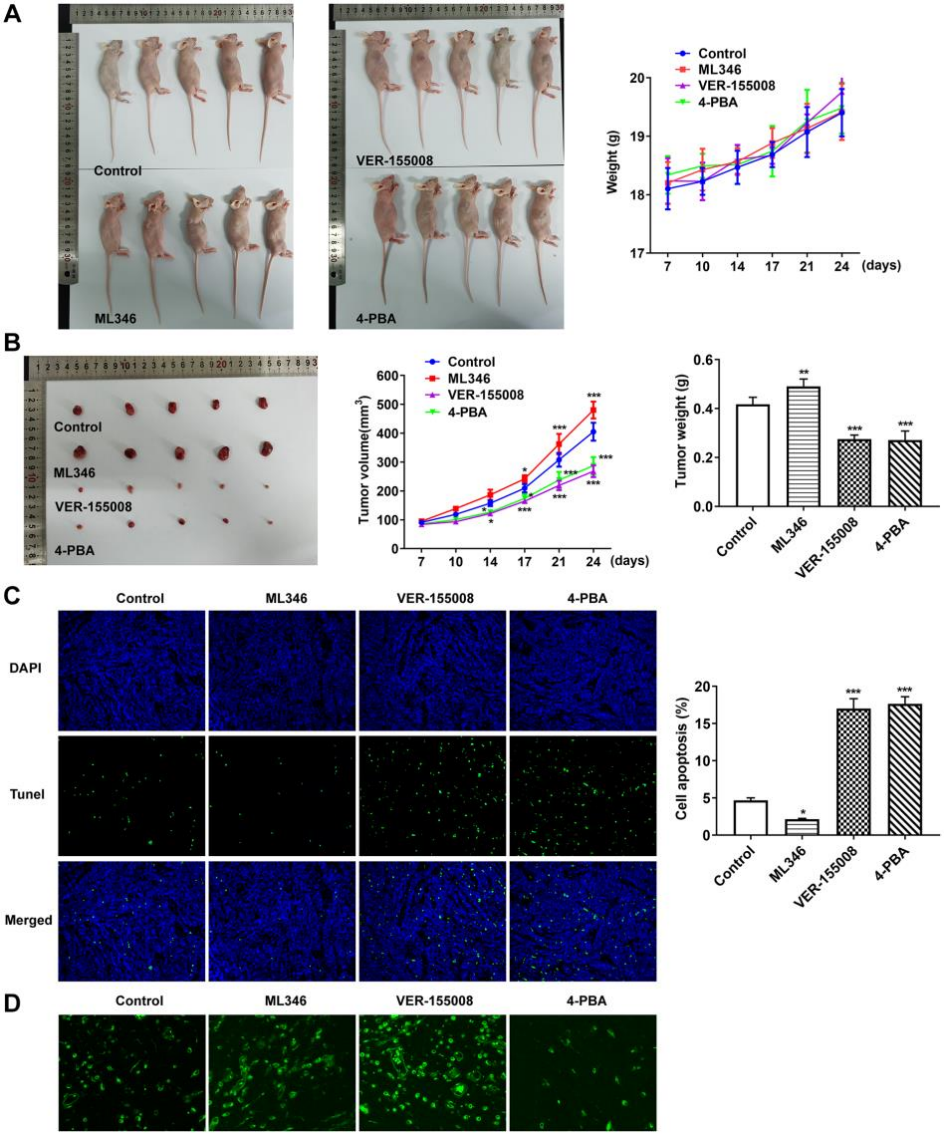
**Inhibition of HSP70 and ERS could significantly promote tumor apoptosis and inhibit tumor growth in the absence of pepsin stimulation.** (**A** and **B**) Tumor photographs and tumor volume and weight in nude mice with LC. (**C**) TUNEL assay detected apoptosis. (**D**) The autophagy detection kit measured autophagy levels. ^*^p < 0.05, ^**^p < 0.01, ^***^p < 0.001 vs. Control.

**Figure 8:**
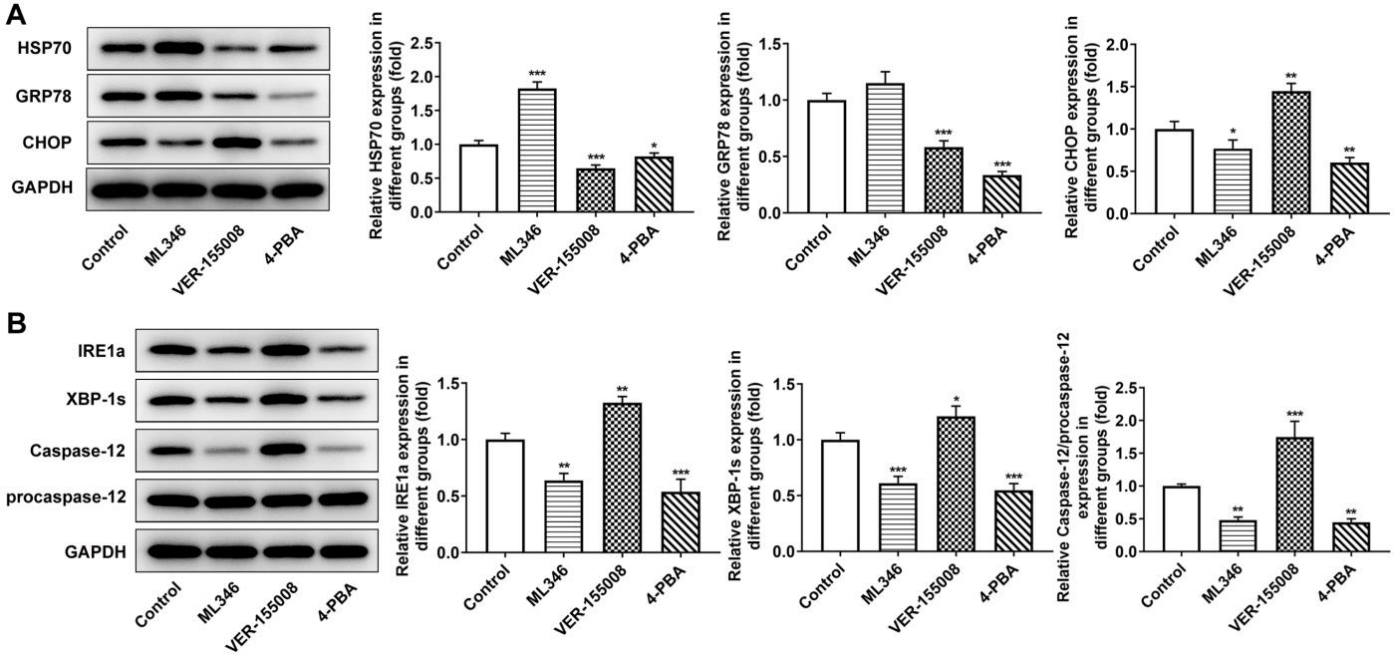
**The effect of inhibition of HSP70 and ERS on ERS-related proteins in tumor-bearing mice.** (**A** and **B**) Western blot detected the expression of ERS-related proteins. ^*^p < 0.05, ^**^p < 0.01, ^***^p < 0.001 vs. Control.

In the above experiments, the influences of HSP70 interference on ERS, apoptosis and autophagy in normal cells and LC cells under pepsin stimulation were examined. Subsequently, in animal experiments, HSP70 activator ML346, inhibitor VER-155008 and ERS inhibitor 4-PBA were applied in murine model bearing LC to explore the effects of HSP70 on tumor growth and ERS without pepsin stimulation. The weight and volume of tumors were measured and photographed. Compared with the control group, the tumor size and weight in ML346 group were larger and heavier, the tumor size and weight in VER-155008 group was smaller and lighter, and the tumor size and weight in 4-PBA group was also smaller and lighter (Figure 7A and 7B).TUNEL staining was used to detect the apoptosis in tumor tissues. Compared with the control group, apoptosis was decreased in ML346 group, and significantly increased in VER-155008 and 4-PBA group (Figure 7C). Autophagy was detected by the autophagy kit, and the results showed that compared with the control group, autophagy was increased in the ML346 and VER-155008 groups, and decreased in the 4-PBA group (Figure 7D). These results indicated that inhibition of autophagy or excessive autophagy might cause adverse effects on tumor tissues. Western blot results showed that compared with the control group, ML346 apparently enhanced HSP70 expression and declined CHOP expression, while exerted no statistical influence on GRP78 expression. The addition of VER155008 prominently down-regulated HSP70 and GRP78 expression while up-regulated CHOP expression. 4-PBA led to the decrease on HSP70, GRP78 and CHOP expression (Figure 8A). Compared with the control group, the expression of IREα, XBP-1S and procaspase-12 was significantly decreased in ML346 and 4-PBA groups, while increased in VER-155008 group (Figure 8B).

## DISCUSSION

HSP70 is a highly evolutionarily conserved family, and it is also the most thoroughly studied family so far, especially in terms of the apoptosis pathway [17, 18]. Notably, the decrease on HSP70 induces cancer cell death, demonstrating that death stimulation can induce a protective response in cells and that HSP70 has important anti-apoptotic properties [17]. HSP70 is rapidly expressed in a variety of stress states, such as UPR induced by the accumulation of misfolded proteins, and ERS [19]. Current studies believe that from the perspective of the mechanism of action, the increase of HSP70 actually protects against the stress in the body and cells. For example, during heat stress, coenzyme Q10 protects primary myocardial cells by up-regulating HSP70 expression [20]. In the mouse model of retinal degeneration, the expression of endogenous HSP70 is briefly elevated at the early stage and then sharply decreased with cell death, suggesting that this is the initial adaptive response of HSP70 to cellular stress [21]. In addition, the continuously increased expression of HSP70 is a feature of many tumor cells, and the increased reactivity of HSP70 can promote the folding of cancer-related proteins, promote the activity of tumor cells, inhibit the apoptosis of tumor cells, and have a significant protective effect on tumor cells [22]. In our experiment, we found that HSP70 was increased in pepsin-stimulated laryngeal epithelial cells and LC cells, thereby inhibiting ERS and ERS-induced apoptosis and autophagy. In our experiment, we also found that normal cells were more sensitive to pepsin stimulation than cancer cells.

On one hand, the increase of HSP70 can enhance the degradation of unfolded protein. On the other hand, it can also induce autophagy to an appropriate extent and enhance the survival ability of cells in adverse environment [23]. However, if the UPRs are too strong or too long for the ERS to regulate themselves, apoptotic pathways in the downstream of ERS will trigger apoptosis, and ERS-associated autophagy will no longer be a “protective” effect [24–26]. In the experiment, we found that under the background of pepsin stimulation, inhibition of HSP70 could reduce the expression of GRP78 in normal cells and LC cells, and only inhibited downstream apoptosis-related pathways in laryngeal epithelial cells instead of in LC cells. It was speculated that tumor cells themselves had certain ERS and apoptosis levels, and were less sensitive to pepsin stimulation and HSP70 inhibition. In addition, normal cells and cancer cells had different sensitivity to pepsin and ERS due to different environments, leading to different trends under HSP70 intervention. In view of the opposite treatment strategies of normal cells and cancer cells, we believed that inhibition of HSP70 could inhibit the apoptosis of normal cells and might not significantly inhibit the apoptosis of cancer cells. Therefore, the significance of HSP70 expression in the clinical treatment of cancer needs to be judged according to different disease types.

Moreover, HSP70 itself can activate ERS to some extent, and inhibit CHOP and apoptosis, which is contradictory [16]. Therefore, functional changes caused by expression changes of HSP70 are different in normal cells and cancer cells under different environmental backgrounds. HSP70 itself inhibits apoptosis and therefore it cannot be assumed that the apoptosis level will be increased after inhibition of HSP70. In cancer cells, pepsin stimulation increased ERS and HSP70. At this time, inhibition of HSP70 might indeed play a role in promoting apoptosis. But inhibition of HSP70 could also inhibit ERS, which could also inhibit apoptosis phenotype. Combined with these two trends, whether apoptosis was decreased or increased depended on the sensitivity to pepsin stimulation and ERS levels and changes.

Finally, in animal experiments, with the absence of pepsin, the results showed that inhibition of HSP70 and ERS could significantly promote tumor apoptosis and inhibit tumor progression in the tumor environment without ERS load. On one hand, we further verified the regulatory effect of HSP70 on ERS and apoptosis. On the other hand, it was also suggested that stress induced by pepsin stimulation might be a factor affecting the sensitivity of normal cells and cancer cells to drugs. However, due to the limitation of experimental conditions and time, no more detailed control experiments were conducted to verify this, which is the direction of our further study.
